# Canine Parvovirus is diagnosed and neutralized by chicken IgY-scFv generated against the virus capsid protein

**DOI:** 10.1186/s13567-020-00832-7

**Published:** 2020-09-03

**Authors:** Shikun Ge, Long Xu, Ben Li, Fagang Zhong, Xiang Liu, Xiaoying Zhang

**Affiliations:** 1 Chinese-German Joint Laboratory for Natural Product Research, Key Laboratory of Biological Resources and Ecological Environment of Qinba Areas, School of Biological Science and Engineering, Shaanxi University of Technology, Hanzhong, China; 2 Department of Biology, Centre of Molecular and Environmental Biology, University of Minho, Campus de Gualtar, 4710-057 Braga, Portugal; 3 Department of Biomedical Sciences, Ontario Veterinary College, University of Guelph, Guelph, ON Canada; 4 State Key Laboratory for Sheep Genetic Improvement and Healthy Production, Xinjiang Academy of Agricultural and Reclamation Science, Shihezi, China

**Keywords:** IgY-Technology, infectious animal diseases, virus diseases, parvovirus, phage display system

## Abstract

Canine parvovirus (CPV) can cause acute and highly contagious bloody enteritis in dog. To obtain antibodies against CPV, hens were immunized with virus-like particles (VLP) of CPV-VP2. The IgY single chain fragment variables (scFv) were generated by T7 phage display system and expressed in *E. coli* system. The titer of the primary scFv library reached to 1.5 × 10^6^ pfu/mL, and 95% of the phages contained the target fragments. The CPV-VLP and CPV-VP2 protein showed similar reaction values to the purified scFv in the ELISA test, and the results of ELISA analysis using IgY-scFv toward CPV clinical samples were consistent with commercial immunochromatographic assay (ICA) and PCR detection, the scFv did not show cross reactivity with canine distemper virus (CDV) and canine coronavirus (CCV). IgY-scFv was successfully expressed in CRFK cells, and in the virus suppression assay, 55% of CPV infections were eliminated within 24 h. Docking results demonstrated that the number of amino acids of the binding sides between scFv and VP2 were AA37 and AA40, respectively. This study revealed the feasibility of a novel functional antibody fragment development strategy by generating diversified avian IgY-scFv libraries towards the pathogenic target of interest for both detection and therapeutic purposes in veterinary medicine.

## Introduction

Canine parvovirus (CPV) was first identified in 1978 [1]; it has a single-stranded DNA genome of negative polarity, about 5200 bp in length. The CPV capsid is a 25 nm diameter icosahedron containing three structural viral proteins (VP1, VP2 and VP3) and two non-structural proteins (NS1 and NS2), among them, VP2 accounts for 90% of the viral capsid and represents the major determinant of host range and virus-host interactions [2]. Although the conventional attenuated and inactivated CPV vaccines have been successful in reducing the disease outbreaks, the genus of parvovirus still causes severe epizootics worldwide and leads to severe economic losses in dogs [3].

Antibody based approach is promising in CPV disease diagnosis, therapy and prevention, and the relevant hyper immunoglobulin G (IgG) and full-length monoclonal antibody (mAbs) have been routinely applied in the veterinary practices [4]. At present, most commercially available mAbs are produced in mammalian system using hybridoma technology. Functional antibody fragments (i.e.: scFv, Fab) generated by phage-display technology have been not yet routinely evaluated and applied in the veterinary medicine despite it has been well confirmed that such engineered antibodies offer a series of advantages over polyclonal and full-length monoclonal antibodies. Antibody phage display technology is to display polyclonal antibodies on the surface of the phage shell protein and to screen the specific monoclonal antibodies by bio-panning procedure [5]. Phages are more stable and can be stored for years at 4 °C; it can be re-produced rapidly, successively and inexpensively with a confirmed sequence in the prokaryote or eukaryote systems without hybridoma and immunization procedure. Furthermore, higher affinity mutants of scFv can be generated through site directed mutagenesis which is much easier and simpler to be performed [6].

Chicken (*Gallusb gallus domesticus*) IgY antibody generated by IgY-Technology is another conventional approach in generating large amount of high specific antibody and have been used for broad biomedical purposes owning to a series of advantages such as higher productivity, better animal welfare, higher immunogenicity to mammal conserved proteins and lower cross-reactivity, as compared to the generation and application of mammalian serum IgG [7]. Our previous work confirmed that the generated polyclonal IgY targeted to CPV-VP2 could apply into the immunotherapy and immunoprophylaxis for the CPV infection [8]. In recent years, as an interesting tendency in IgY technology, generation of IgY-scFv in order to better combine the biological superiorities of both IgY and recombinant antibody fragment, is gaining increasing attention and application [9, 10]. Our previous studies demonstrated that IgY-scFv can be generated and applied in different immunoassays for the detection of small molecules gentamicin [11] and large molecules pIFN-γ [12] in the veterinary practice.

As an attempt to understand the feasibility of chicken IgY-scFv in diagnosis and therapy of veterinary diseases, this study aimed to construct and characterize chicken sourced scFv against CPV-VP2 virus like particles (VLP) using phage display technology, and to evaluate the specificity, sensitivity and virus inhibition ability of the obtained scFv.

## Materials and methods

### Immunization of chicken

Twelve-week old white Leghorn hens were immunized intramuscularly with CPV-VLP (provided by Dr. Shiqi Sun [13]) mixed with Freund’s adjuvant (Sigma-Aldrich, St. Louis, MO, USA) at four different sites of breast muscles. CPV-VLP protein (125 µL, 2 mg/mL) in equal volume of phosphate buffered solution (PBS, 0.01 M, pH 7.4) was emulsified with Freund’s complete adjuvant (FCA; Sigma-Aldrich, St. Louis, MO, USA) in the first immunization, and four booster immunizations were followed up with Freund’s incomplete adjuvant (FIA; Sigma-Aldrich, St. Louis, MO, USA) at 2-week intervals. All experimental animal protocols were reviewed and approved by the institutional Ethics Committee for the use of laboratory animals.

### Construction of anti-CPV-VLP scFv

The hen’s spleen was collected to extract the total RNA by Total RNA Kit (Tiangen Biotech, Beijing, China), and the first-strand cDNA was synthesized by HiScript Q Select RT SuperMix for PCR (+gDNA wiper, Vazyme Biotech, Nanjing, China). The heavy variable fragment (V_H_) and light chain variable fragment (V_L_) genes were amplified by PCR with primers (Table 1). The V_H_ and V_L_ were assembled with primers HF-*Eco*R I & LR-*Hin*d III by Overlap PCR. The products of overlap PCR (scFv) were purified through Gel Extraction Kit (Omega, Norcross, GA, USA). This protocol was performed as described in [14].

**Table 1:** **Primers used for PCR.**

Name	Primer sequences (5′-3′)	Application
HF-*Eco*R I	CGGAATTCGGCCGTGACGTTGGACG	V_H_
HR-Linker	CAGAGCCACCTCCGCCTGAACCGCCTCCA	V_H_
	CCGGAGGAGACGATGAC	
LF-Linker	TTCAGGCGGAGGTGGCTCTGGCGGTGGCG	V_L_
	GATCGGCGCTGACTCAGCCGTCCT	
LR-*Hin*d III	AATAAGCTTACCTAGGACGGTCAGGG	V_L_

### Construction of library

The scFv gene products were digested with *Eco*R I and *Hin*d III restriction enzyme and ligated to T7 select 10-3b (0.04 pmol; Novagen, Darmstadt, Germany) by T4 DNA Ligase (TaKaRa Biotechnology, Dalian, China) in a work volume of 5 µL at 16 °C overnight. The ligation products were directly added to T7 package extract (25 µL) and the mixtures were incubated at 22 °C for 2 h in vitro to create the primary library by add Luria–Bertani medium (270 µL) to stop the reaction. The primary scFv library was amplified by liquid lysate amplification refer to the T7 select system manual. The titers of the primary and amplified library were evaluated by plaque assay. This protocol was performed as described in [14].

### Bio panning

The amplified library was subjected to four rounds of bio panning on microplates for the enrichment of the specific scFv phages refer to the T7 select system manual. The microplates were coated with CPV-VLP overnight (15 µg, 10 µg, 8 µg and 5 µg per well in each round) at 4 °C, and washed with Tween-PBS (PBST, tween 0.5%; 150 µL/well) and PBS, respectively. After blocking with skimmed milk (5%) in PBST, the amplified phages from the initial library of each round of panning were added to the microplates and incubated at 37 °C for 1 h, and the bound phage was eluted with SDS (1%) in each round. In the first three rounds, eluted phages were amplified by infect BL5403 bacterial culture (Novagen, Darmstadt, Germany). The enrichment of specificity was determined from the input/output ratio of the phage. A total of random clones of 100 on the plates were selected from the fourth round to identify the positive rate by using a pair of universal primers (Table 1). The amplified library was added with 1% chloroform and stored at 4 °C. This protocol was performed as described in [14].

### Expression and purification of scFv

The phage-scFv with the highest affinity was selected to ligate with pET-30a (+) vector use T4 DNA Ligase (TaKaRa Biotech, Dalian, China). The recombinant plasmids were transformed into BL21 (DE3) competent cells, and the scFv expression was induced with isopropyl-β-D-thiogalactopyranoside (IPTG). The bacterial cells were collected by centrifugation and sonication, and the supernatants and pellets were used for SDS-PAGE analysis, respectively. Protein was purified by HisTrap HP histidine-tagged protein cultured columns (GE, Pittsburgh, PA, USA). This protocol was performed as described in our previous reports [15].

### Identification of scFv

The CPV-VP2 proteins were separated by SDS-PAGE (5% spacer gel and 12% separation gel) and blotted on nitrocellulose membranes (6 cm × 4 cm, 0.45 µm; Schleicher & Schuell, Atlanta, GA, USA). Membranes were blocked with 5% (w/v) nonfat dry milk (NDM) in Tris-buffered saline-Tween (TBS-T; pH 7.4, 0.1%) at room temperature (RT) for 30 min. Membranes were incubated with 12.5 mL TBS-T containing 1% NDM and 5 µg prepared scFv proteins at RT under agitation for 1 h. Membranes were washed with TBST (3 times × 10 min), and incubated with 12.5 mL TBS-T containing 1% NDM and mouse anti-His monoclonal antibody conjugated with HRP (1:5000; CWBio, Beijing, China) at RT under agitation for 30 min. Membranes were washed with TBST (3 times × 15 min), and developed using DAB chromogenic solution detection reagents (Boster, Wuhan, China). Western blot was performed as described in [16].

### Analysis on scFv sensitivity by ELISA

The wells of a 96-well Maxisorp microtiter plate (Nunc, Roskilde, Denmark) were coated with different amounts of CPV-VP2 (0, 0.2, 0.5, 2, 6, 8, 10 ng/µL) dissolved in carbonate buffer (15 mM Na_2_CO_3_, 35 mM NaHCO_3_, 0.001% phenol-red, pH 9.6) and incubated over night at 4 °C. The wells were rinsed with PBST and incubated with scFv protein (different amount) diluted in PBST for 1 h. The wells were rinsed with PBST (3 times × 5 min) and incubated 1 h at RT with mouse anti-His monoclonal antibody conjugated with HRP (1:5000; CWBio, Beijing, China) followed washes with PBST (3 times × 5 min). The color developed using 3, 3′, 5, 5′ tetramethylbenzidine (TMB; Promega Biotech, Beijing, China) for 10 min and the absorbance was read at 450 nm.

### Detection of clinical samples

To determine the accuracy of scFv, a total of 28 clinical dog stool samples were collected using sterile swabs in an animal hospital (Xinger, Xi’an, China). Among them, 24 samples were confirmed CPV positive and 4 were negative by the hospital using commercial colloidal gold test strip (ICA). The samples were homogenized (10%, w/v) in PBS (1 mL, pH 7.2) and centrifuged, the supernatants were used for indirect ELISA and PCR. In order to verify whether scFv has specificity in clinical practice detection, Canine distemper virus (CDV) and coronavirus (CCV) were used to detect the cross-reaction by ELISA.

### Immunocytochemistry Assay (ICA)

The CRFK cells (LMAI Bio, Shanghai, China) were seeded into 6-well plates with optimum culture medium and transfections were performed when the cell monolayer density reached 70%. The transfection mixture (containing the pcMV-3-scFv) was added into each well and incubated for 4 h at 37 °C in moist 5% CO_2_. At 48 h post-transfection, the cells were fixed with paraformaldehyde (4%), permeabilized with Triton X-100 (0.5%) and incubated with BSA (Beyotime, Shanghai, China) to block nonspecific binding sites. They were incubated with diluted His-tag antibody (100 µL; Affinity Biosciences, OH, USA) for 2 h then washed with PBS. Goat anti-Mouse IgG (H + L) Fluor 594-conjugated (1:800; Affinity Biosciences, OH, USA) was added. The stained cells were visualized by use a Nikon Eclipse 80i fluorescence microscope (Nikon, Sendai, Japan). Total protein of the cells transfected 48 h were extracted for western blot; His-tag antibody and Goat anti-Mouse IgG HRP (Biosharp, Hefei, China) were used as primary and secondary antibodies, respectively. ICA was performed as described in [17].

### Measurement of virus growth curve

Normal CRFK cells (10^5^/mL) were seeded into 96-well plates, and the virus infection was performed when the cell density reached 70%. The viral fluid was serially diluted in tenfold, added to a 96-well plate, and 8 wells were repeated for each virus concentration. The 96-well plate was placed in 37 °C at 5% CO_2_ incubator, and the tissue culture infective dose (TCID_50_) was determined after the number of pathological holes was determined.

ScFv Transfected CRFK cells and normal cells were seeded into 96-well plates, and the cells were infected with CPV (CPV-HY strain offered by Century Yuanheng Animal Epidemic Prevention Technology Co., Ltd., Beijing, China) in 0.1 MOI when the cell monolayer density reached 100%. After 1 h, the supernatant virus solution was removed and replaced with a cell maintenance solution. The cell supernatants were collected at 2 h, 4 h, 8 h, 12 h, 24 h, 36 h, 48 h, 60 h, 72 h and 84 h, respectively, and the TCID_50_ values of each time point were measured to plot the growth curve.

### ScFv-VP2 docking

Docking was performed by Discovery Studio 4.5 software (BIOVIA, San Diego, CA, USA) and the Antibody Model Cascade program in Discovery Studio software was used to build the molecular model of the scFv. Then, to obtain the docking conformation, the scFv molecular model was docked with VP2 using the ZDock molecular dock program. Finally, the Residues Contact Frequency (RCF) algorithm was used to analyze the predicted results, and the bind surfaces of amino acids in scFv and VP2 were obtained.

## Results

### Construction of scFv library

**Figure 1:**
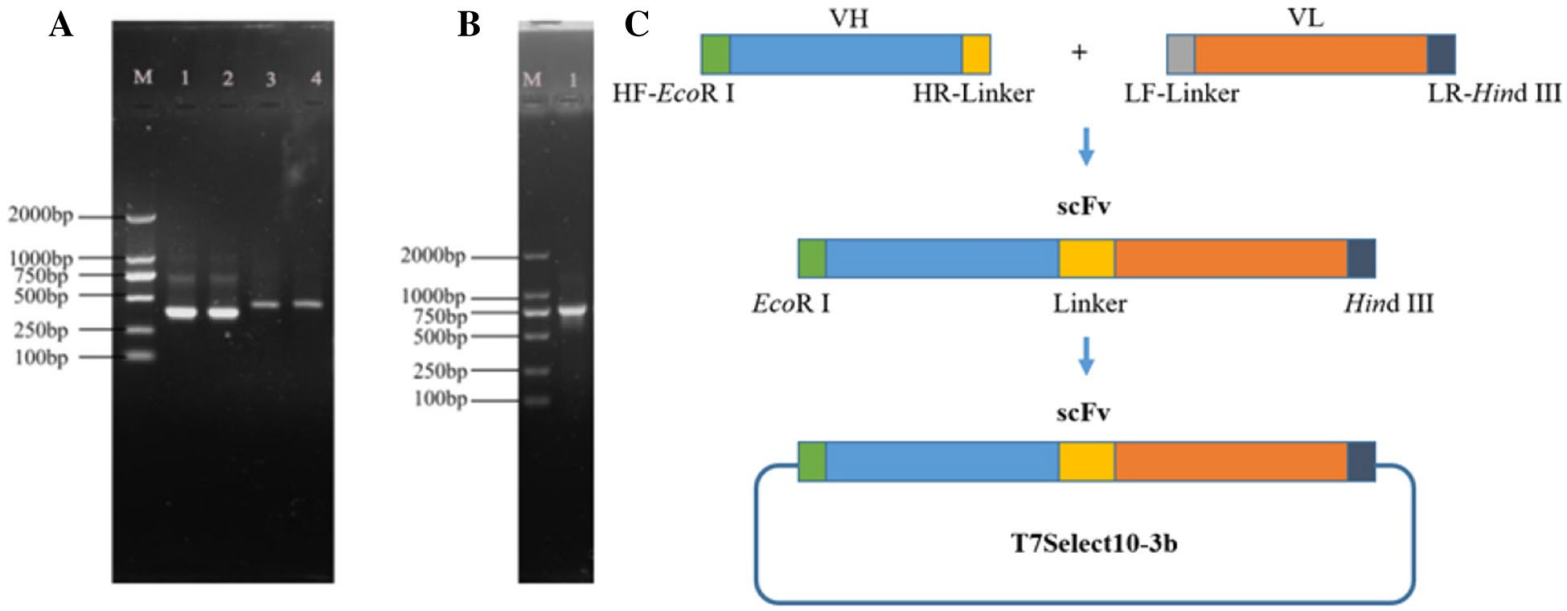
**Construction of the scFv antibody phage display library. A** PCR products of VL (370 bp, lane 1 and 2) and VH (420 bp, lane 3 and 4); **B** PCR products of scFv, 800 bp (lane 1). M: marker; **C** schematic diagram of scFv construction.

The lengths of VH-linker, VL-linker and scFv were approximately 370 bp, 420 bp and 800 bp, respectively (Figures 1A and B). The titer of the primary anti-CPV scFv library (Figure 1C) and the amplified library were 1.5 × 10^6^ pfu/mL, and 8.2 × 10^11^ pfu/mL, respectively. There were 95% of the phages containing the target fragments in the primary scFv library.

### Screening of scFv phage library

**Figure 2:**
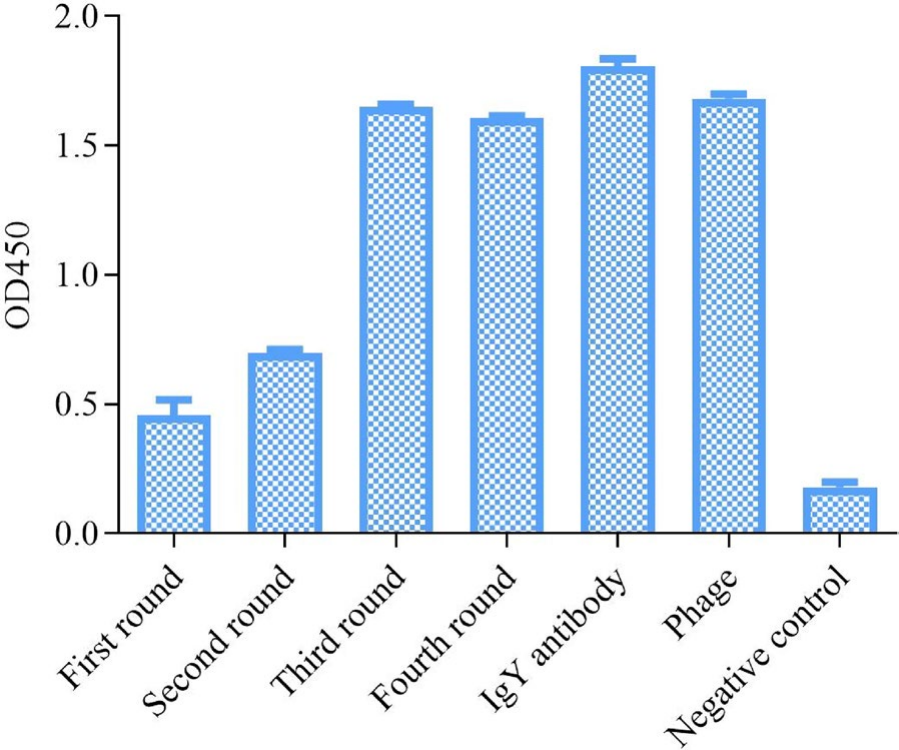
**Phage-ELISA.** Notes: Samples from round 1-4: Phage in each of 4 rounds of bio-panning procedure; IgY antibody sample: IgY polyclonal antibody against CPV-VLP extracted from egg yolk; Phage sample: The primary scFv phage library; Negative control sample: The negative control sample was PBS

**Figure 3:**
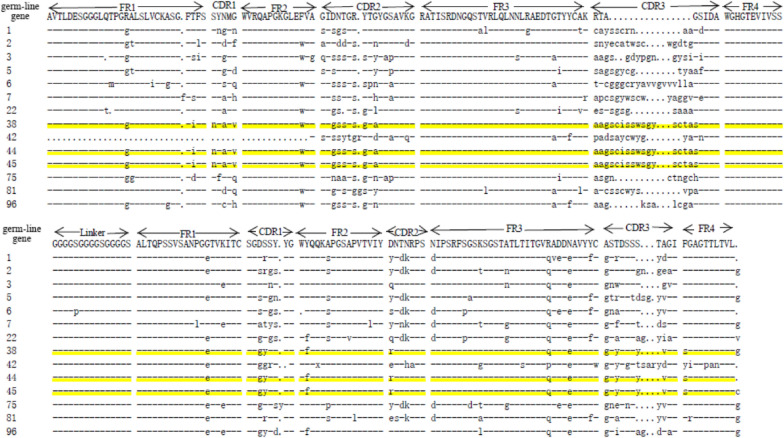
**scFv sequence analysis.** The yellow markers represented the same sequences.

After 4 rounds of “bind-elute-amplify” bio-panning procedure, the data of input and output phages in each round indicated that the phage library had been enriched 100 times (Figure 2 and Table 2). A total of 15 scFv genes showed relatively high binding capacity to CPV-VLP in the reactivity detection of 100 phages, and the sequencing confirmed that all the 15 scFv genes have complementary determining region 3 (CDR3) that was the main mutation region in both VH and VL. There were few clones having limited mutations in framework region (FR, Figure 3). The phage-scFv (No. 96) showed the highest binding force was chosen for further experiments.

**Table 2:** **Library size and phage titer of each panning.**

Round	Coating concentration (per well)	Input (pfu/mL)	Output (pfu/mL)	Amplification library (pfu/mL)
1	15 µg	1 × 10^12^	7.2 × 10^6^	4.95 × 10^12^
2	10 µg	1 × 10^11^	4.64 × 10^7^	1.31 × 10^13^
3	8 µg	1 × 10^11^	8.8 × 10^8^	2.8 × 10^12^
4	5 µg	1 × 10^11^	1.52 × 10^8^	–

### ScFv expression and purification

**Figure 4:**
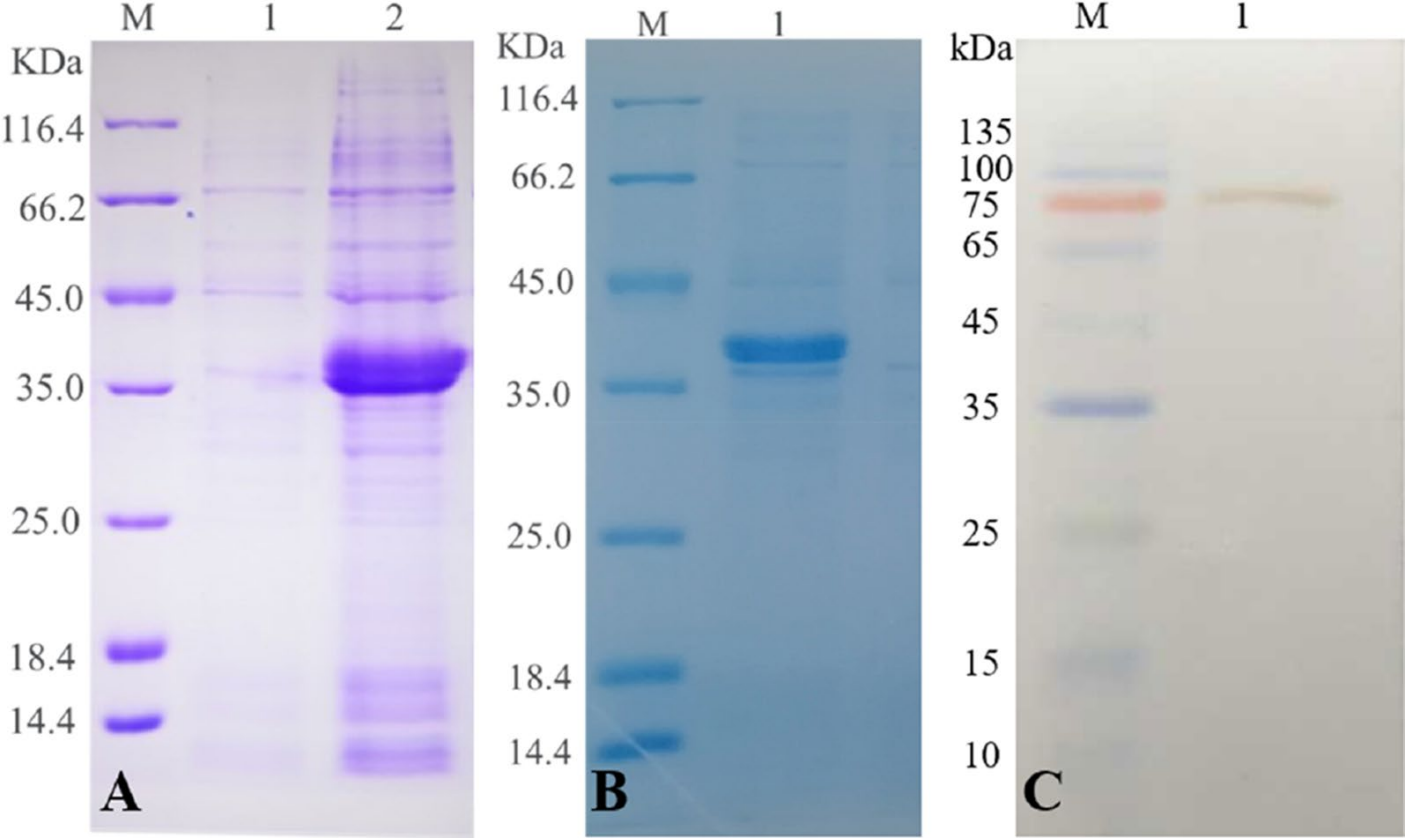
**SDS-PAGE and western blot analysis of the obtained scFv. A** lane 1, *E. coli* supernatant; lane 2, *E. coli* precipitation; **B** lane 1, the renaturation of scFv protein; **C** lane 1, antigen was CPV-VP2 protein, incubated with primary antibody scFv.

The solubility analysis showed that scFv mainly existed in the form of inclusion body (38 kDa; Figure 4A, lane 2). The denaturation and purification of scFv inclusion body protein showed a single protein band with high purity (Figure 4B). The VP2 protein could bind to scFv with a single binding strip (85 kDa; Figure 4C).

### ScFv sensitivity analysis

**Figure 5:**
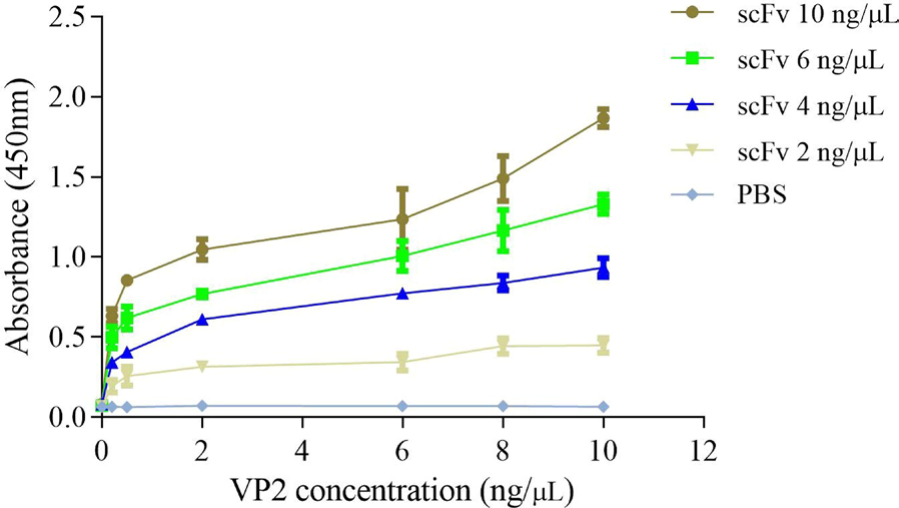
**Antigen-binding analysis by ELISA.** scFv in different concentrations were added to 96-well plates coated with soluble VP2 in different concentrations (0, 0.2, 0.5, 2, 6, 8, 10 ng/µL). Binding was detected with mouse anti-His monoclonal antibody conjugated with HRP. The binding activity was measured as absorbance at 450 nm produced by peroxidase. Data of each point represented as Mean ± SD of triplicate.

The immunoreactivity of scFv against soluble VP2 was examined by ELISA. scFv bound in a dose-dependent manner to the soluble VP2 (Figure 5). The minimum antibody concentration for the detected antigen was 2 ng/µL.

### Specificity and cross-reactivity of IgY-scFv in CPV clinical sample tests

**Figure 6:**
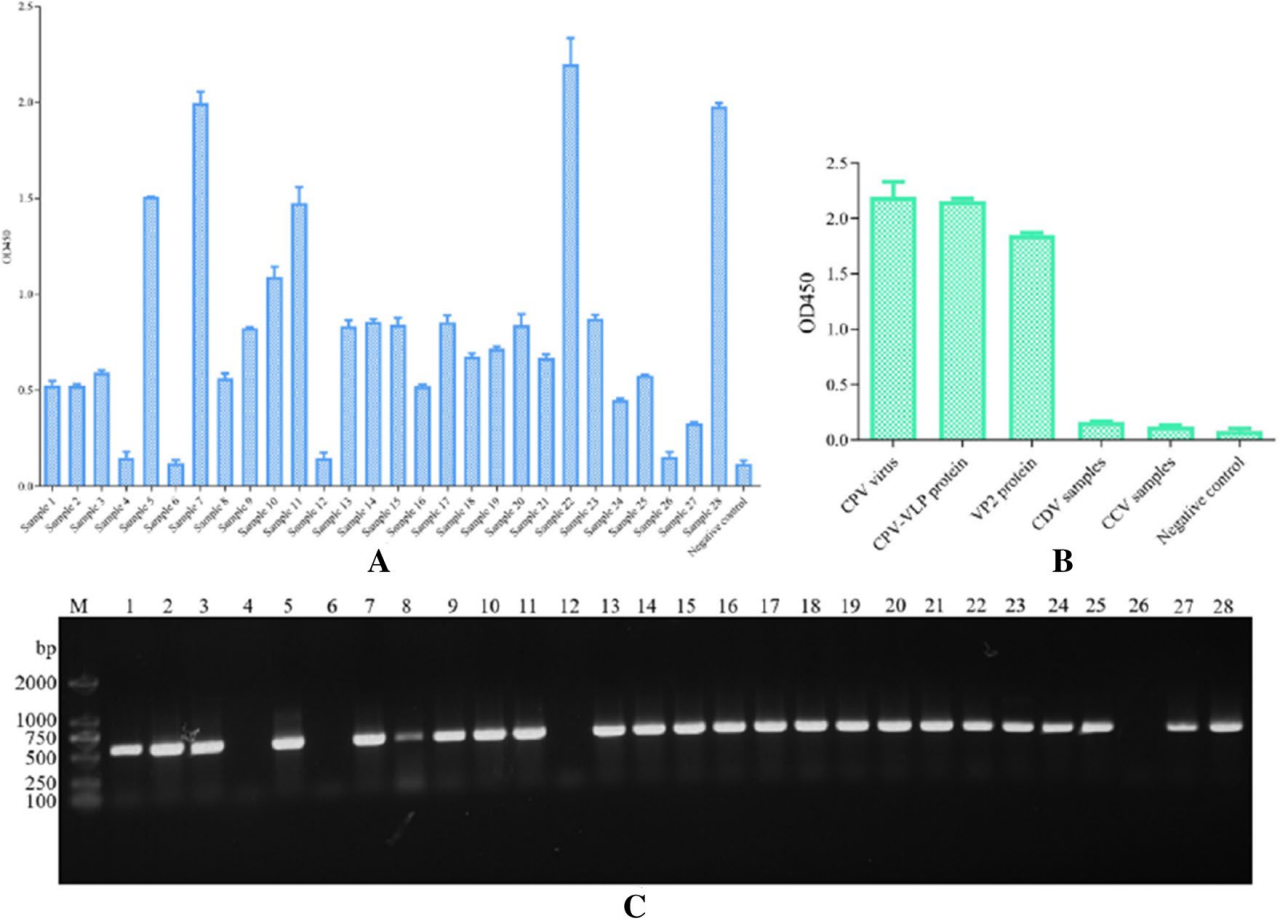
**Analysis on CPV clinical samples.** Clinical samples of CPV were analyzed by ELISA (**A**), PCR (**C**) and ICA (data not shown); the cross reactivities of anti-CPV-IgY-scFv with CDV and CCV were analyzed by ELISA (**B**). CPV was CPV-HY strain; VP2 was expressed in the prokaryotic system.

The coincidence of ELISA (Figure 6A) and PCR (Figure 6C) with ICA (data not show) was 100% and 85.7%, respectively. The scFv showed no cross reactivity with CDV and CCV (Figure 6B).

### Neutralization effect of scFv to CPV in CRFK cells

**Figure 7:**
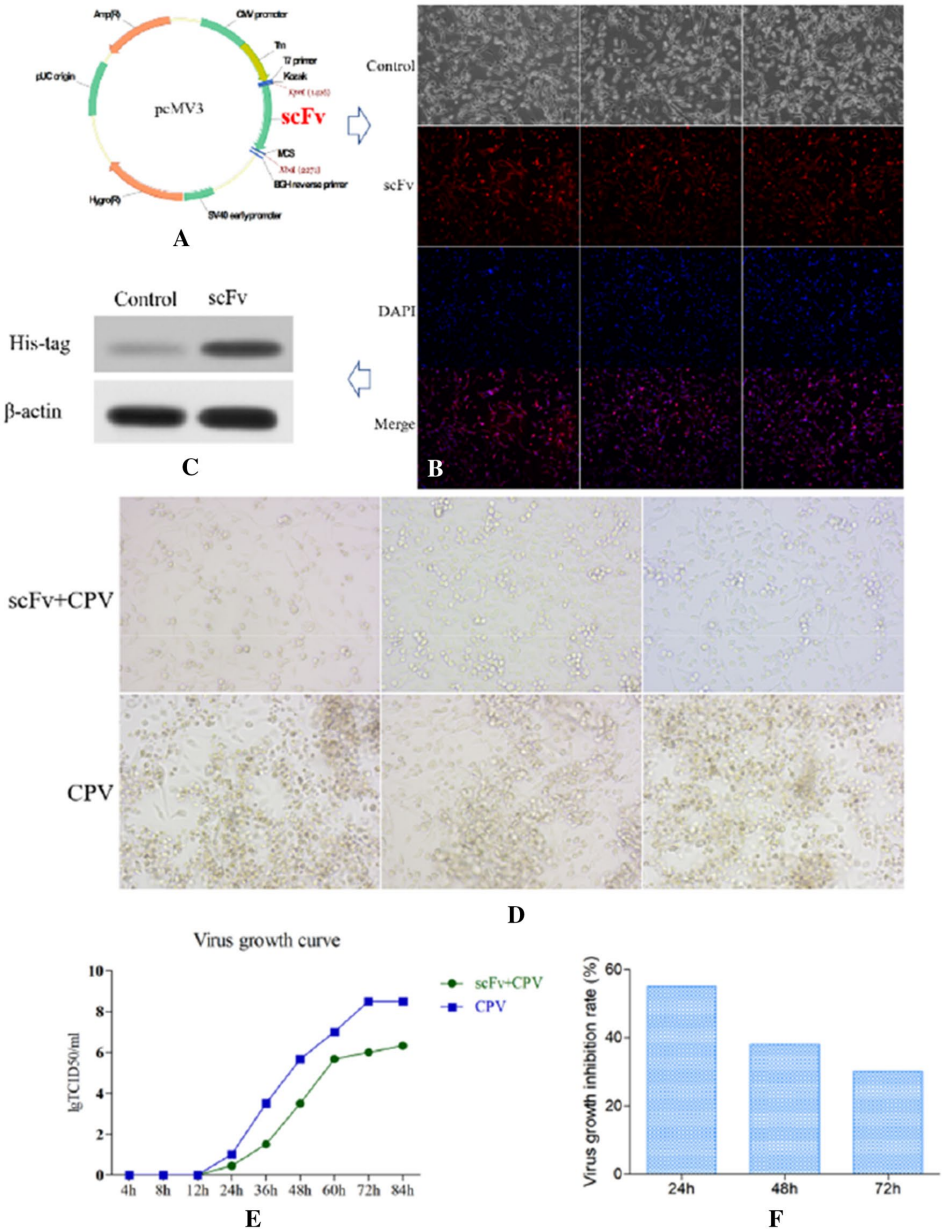
**Neutralization effect of obtained scFv to CPV. A** scFv gene was ligated pcMV-3 vector. **B** Immunofluorescence analysis of scFv transfected CRFK cells. control: CRFK cells of pcMV-3 without scFv, scFv: CRFK cells of pcMV-3 with scFv, DAPI: staining nuclei. **C** western blot detection of scFv transfected CRFK cells, control represent empty pcMV-3 transfected CRFK cells. **D** after transfecting the cells with scFv for 24 h, the cells were infected with CPV (MOI = 0.1). **E** growth curve of CPV in CRFK cells with scFv and without scFv expression. **F** The proportion of TCID_50_ of virus in different cell treatment groups at different time points.

The sequencing results showed that the scFv and pcMV-3 vectors were successfully connected (Figure 7A), IFA confirmed that scFv was significantly expressed in CRFK cells (Figure 7B). Immunoblotting further confirmed that the scFv protein was correctly folded and modified in the cell (Figure 7C). The CRFK cells expressing scFv showed a small amount of cytopathic effect (CPE) after CPV infection; the cells without scFv expression were significantly broken away from the bottom wall, became round, and some cells even broke up (Figure 7D). Virus TCID_50_ at different time points was determined; the growth rate of virus in cells expressing scFv was significantly lower than that in cells not expressing scFv (Figure 7E). The inhibition rates of scFv on virus growth at 24 h, 48 h, and 72 h were 55%, 38% and 30%, respectively (Figure 7F).

### The binding sides of the scFv-VP2

**Figure 8:**
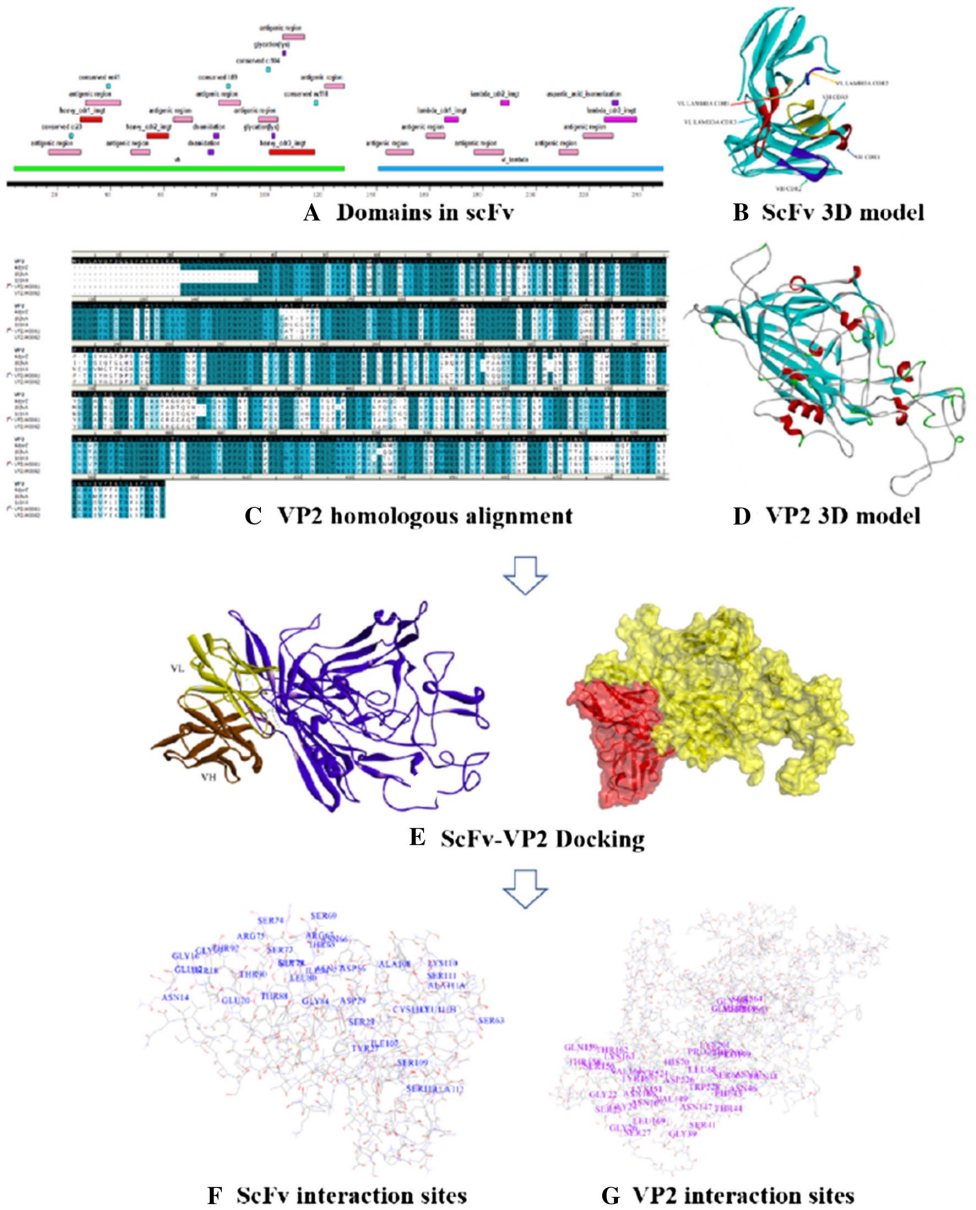
**Docking analysis on VP2-scFv binging.**

The main structural domains of scFv were VLCDR1, VLCDR2, VLDR3, VHCDR1, VHDCR2, and VHCDR3, with 13 antigen-binding sites on scFv (Figure 8A). The 3 dimensional scFv model was subsequently constructed (Figure 8B). A total of 5 highly similar VP2 homologous sequences were simulated by software (Figure 8C), the VP2 molecular stereo model was established by combining the characteristics of each sequence (Figure 8D). After analysis on scFv and VP2 binding mode (Figure 8E), the interacting amino acids at the binding sides of scFv (AA37) and VP2 (AA40) were confirmed (Figure 8F and G).

## Discussion

The mAbs have been widely applied in the biomedical areas owning to their high specificity and homogeneity. However, mAbs produced by mammals may have the side effects of immunogenicity, thrombocytopenia and hypersensitivity reactions, etc., which has greatly limited their application in target detection and treatment [14, 15]. With the development of antibody library technology and humanized antibody modification technology, humanized recombinant antibodies have been gradually developed and entered clinical trials [16]. Diversified antibody generation strategies could be a future tendency in antibody engineering in order to better combine the characteristics and advantages of antibodies from different sources. As a notable example, Brolucizumab (Beovu) is the first FDA approved rabbit-derived scFv used as vascular endothelial growth factor (VEGF) inhibitor for the treatment of exudative (wet) age-related macular degeneration (AMD), diabetic macular oedema and macular oedema secondary to retinal vein occlusion, which could better overcome the possible side effects (discomfort and increased tears in the affected eyes, itchy or watery eyes, dry eyes, swelling of the eyelids, etc.) of murine-derived IgG-Fab fragment (Lucentis) [17]. In avian IgY, similar attempts have also been made. For instance, the snake venom contains neurotoxic proteins, the urgent administration of hyperimmune serum from horse used to be the most efficient treatment, which can recognize many different antigenic determinants. However, generation of equine anti- venom is costly and associated with several potential side effects. Chicken IgY-scFv has been generated against glutaraldehyde-attenuated *Daboia russelii formosensis* (DRF) venom proteins for passive immunization, which can identify and neutralize the toxic activity of the venom components, with only small quantity of antigens required to induce a significant antibody response in hens, and can be also used as a rapid diagnostic tool for wound secretions to determine snake types [18].

As summarized by previous authors, owning to their unique structure, phylogenetic distance and tmechanisms of molecular diversification, IgY antibodies provide a series of important advantages over mammal IgG, including the stronger immune responses of chicken system to the proteins conserved among mammals, decreased/no cross-reactivities (i.e.: rheumatoid factor, human anti-mouse IgG antibody, complement system, Fc receptors) in the mammal systems [19, 20], and more convenient design of primer against IgY-scFv (Table 1), as IgY only has one isotype and lacks hinge region [9]. Furthermore, it is noteworthy to address, recent studies confirm that from the glycobiology point of view, recombinant IgY antibody could be a potentially promising immune-therapeutic candidate after proper antibody engineering and expression as IgY is more heavily glycosylated [21], and has higher sialic acid content [22] as compared to mammal IgG. Recombinant functional antibody fragments remove or reduce irrelevant structures, while retain the specificity and main biological activities of natural antibodies, which offers a wider application prospect than natural antibodies [23]. Recombinant IgY-scFv could combine the advantages of both IgY molecular and functional antibody fragment [9].

Designing on chimeric antibody could be the next step for IgY-scFv study in order to provide better compatibility of the antibody in the host system, and to recoup the possibly decreased specificity and affinity of antibody fragments as compared to full length antibody. Recent study confirmed that mammalian IgG and avian IgY shared compatible V-C region interfaces, which may be conducive for the design and utilization of mammalian-avian chimeric Abs [24].

In our study, the high consistency of ELISA analysis to PCR and ICA on clinical samples confirmed the specificity of the obtained IgY-scFv (Figure 6), which offers the potential using IgY-scFv for rapid detection of CPV. scFv neutralized the virus (Figure 7D), inhibited CPV replication with significantly reduced growth rates of the CPV observed in the CRFK cells (Figure 7E, F), which provides the value of further therapeutic investigation of obtained IgY-scFv.

As alternative to viral components, CPV-VP2-VLP was used as immunogen in this study. With increasing applications in vaccine design and immunization, VLP has been recognized as safe and effective particle to stimulate adequate immune responses for both viral and non-viral diseases by inducing lymphocyte proliferation and specific antibody with high titer [25]. The docking of scFv-VP2 shows that there were 13 antigen-binding sites on scFv, with high binding force to VP2. According to Residues Contact Frequency (RCF) algorithm analysis, 37 binding amino acids on scFv and 40 binding amino acids on VP2 were involved in the binding (Figure 8), these results provide us confidence that a well-designed CPV-VLP can be used as a potent immunogen to induce qualified specific antibodies.

In Conclusion, we demonstrated that specific IgY-scFv can be generated with high specificity and significant inhibition to CPV growth. As a preliminary evaluation, our work revealed the potential of IgY-scFv as a novel approach in veterinary diagnosis and therapy.

## Data Availability

All data supporting our findings are included in the manuscript.

## Abbreviations

CCV: coronavirus
CDV: canine distemper virus
CPV: canine parvovirus
ICA: immunochromatographic assay
IgG: immunoglobulin G
IgY-scFv: chicken IgY single chain variable fragments
IPTG: isopropyl-β-d-thiogalactopyranoside
mAbs: monoclonal antibody
scFv: single chain fragment variables
VLP: virus-like particles
